# Rituximab and Fludarabine or Cyclophosphamide Combination Treatment for Older Waldenström Macroglobulinemia Patients

**DOI:** 10.4274/TJH.2013.0030

**Published:** 2013-12-05

**Authors:** XiaoWei Xu, Chun Wang, YaMin Wang, LiLi Zhou, HaiTao Bai

**Affiliations:** 1 Department of Hematology, Shanghai First People’s Hospital, Shanghai, Jiao Tong University, Shanghai, China

**Keywords:** Monoclonal IgM protein, Rituximab, Waldenström macroglobulinaemia

Waldenström macroglobulinemia (WM) is a rare and incurable B cell malignancy. Most WM cells express CD20, which enables the use of anti-CD20 monoclonal antibody rituximab-based strategies. Rituximab has been evaluated in WM for about 10 years as a single agent with major response rates of 30% to 40%, whereas the combination of rituximab with chemotherapy has resulted in response rates of 70% to 90% [1,2,3].

However, the extent to which achievement of complete response (CR) confers clinical benefit is still debatable. As such, we administered rituximab (375 mg/m2, day 0) combined with fludarabine (40 mg, days 1-3) or cyclophosphamide (200 mg, days 1-2) in WM patients naive to rituximab and report herein the toxicities and effects of these combinations. A total of 4 elderly patients (ages older than 80) were identified with WM diagnoses from 2007 to 2012 in our department.

According to recommendations for assessing response from the Third International Workshop on WM, 2 of 4 (50%) patients achieved complete response, 2 (50%) had major or minor response, and the overall response rate was 100% at the end of the treatment [4]. 

The individual changes in serum IgM, IgM paraprotein, and hemoglobin levels for all patients are shown in Figure 1. 

Hematologic toxicity was one of the major side effects of this regimen. Neutropenia and thrombocytopenia were found in 2 patients at the end of the treatment. Pulmonary infection was observed in one patient. 

With a median follow-up of 27 months, all 4 patients died. Two patients with sustainable CR died of pulmonary infection or heart failure, respectively, after follow-up for 11 and 36 months. One patient who achieved a major response duration of 34 months died of duodenal squamous adenocarcinoma. The last patient with minor response had progression of disease in 2 months and died of WM after 10 months of follow-up. 

Gruson et al. assigned a rituximab and fludarabine combination in a treatment cycle that was repeated every month for 6 months. Five patients aged 52-85 years with IgM achieved overall and complete response at 80% and 40%, respectively [5]. Several analyses have identified age as a major adverse factor [6]. Our results showed that 2 patients with sustainable CR were older than 80 years old and had high-risk International Prognostic Scoring System for WM scores. Therefore, with this combination regimen, age is no longer a major risk factor. 

In a study by Dimopoulos et al., which combined rituximab and fludarabine or cyclophosphamide in 11 patients with WM, combination treatment was used monthly for 4 cycles. The partial response rate was only 55% and no patients achieved complete response [7]. The 4-cycle combination therapy was thus not enough for WM patients and 5-6 cycles were necessary. 

In conclusion, rituximab in association with fludarabine or cyclophosphamide is well tolerated and effective for elderly WM patients. 

## CONFLICT OF INTEREST STATEMENT

The authors have declared no conflicts of interest. 

## Figures and Tables

**Figure 1:**
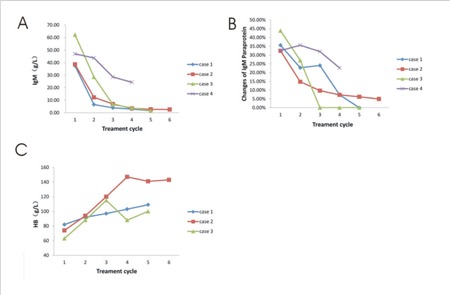
Individual changes in A) IgM, B) IgM paraprotein, and C) HB serum levels
